# The Effect of Feeding Liquid or Dry Creep Feed on Growth Performance, Feed Disappearance, Enzyme Activity and Number of Eaters in Suckling Piglets

**DOI:** 10.3390/ani11113144

**Published:** 2021-11-04

**Authors:** Nanna Byrgesen, Johannes Gulmann Madsen, Christina Larsen, Niels Jørgen Kjeldsen, Malene Skovsted Cilieborg, Charlotte Amdi

**Affiliations:** 1 Department of Veterinary and Animal Sciences, Faculty of Health and Medical Sciences, University of Copenhagen, Grønnegårdsvej 2, 1870 Frederiksberg, Denmark; nanna.byrgesen@gmail.com (N.B.); johannes.g.madsen@sund.ku.dk (J.G.M.); christinalarsen@sund.ku.dk (C.L.); macilie@sund.ku.dk (M.S.C.); 2 SEGES Danish Pig Research Centre, Axeltorv 3, 1609 Copenhagen, Denmark; njk@seges.dk

**Keywords:** dry creep feed, eaters and non-eaters, enzyme activity, feed intake, liquid creep feed

## Abstract

**Simple Summary:**

The transition from sow’s milk to a vegetable-based diet around weaning is one of the most critical periods in a pig’s life, due to the strong association between undigested nutrients and post weaning diarrhoea. For practical and economic reasons, piglets are weaned before their gastro-intestinal tract has fully developed. Early provision of creep feed could be one way of promoting gut maturation and may thus be used as a preventive tool for post weaning diarrhoea instead of medicinal zinc oxide. Increasing the activity of enzymes involved in the digestive processes might enhance gut maturation, thereby preparing the piglet for weaning. Therefore, the objective of this study was to investigate the effect of dry and liquid creep feed on growth performance, enzyme activity and the number of pigs actually consuming creep feed. In conclusion, in this on-farm setup, the pigs fed the dry diet displayed a greater enzyme activity in the proximal part of the small intestine and a higher weight gain, suggesting more mucosal maturation and adaptation to a vegetable-based diet.

**Abstract:**

The aim of this study was to investigate the effect of two dietary treatments (liquid creep feed (LCF) and dry creep feed (DCF)) offered during the suckling period on feed disappearance, number of eaters, and intestinal enzymatic development at weaning in an on-farm study with 347 piglets. Piglets were allocated to either the DCF or LCF treatment from day 10 to day 24 postpartum for 9 h a day. Red ferric oxide (1%) was added to the diet to categorize piglets into eating categories (good eaters, moderate eaters, or non-eaters) via faecal swabs. At weaning, 40 piglets were sampled for intestinal enzymatic development. The LCF treatment increased the dry matter disappearance from day 10–18 (*p* < 0.001). The percentage of good eaters, moderate eaters and non-eaters did not differ between treatments (*p* > 0.05). The DCF pigs displayed greater average daily gain (ADG) pre-weaning (*p* = 0.024), and a greater body weight (BW) at day 61 (*p* < 0.001). The activity of lactase, maltase and sucrase in the proximal part of the small intestine were greatest (*p* < 0.001) in the DCF pigs.

## 1. Introduction

In conventional herds, piglets are weaned abruptly when 3–4 weeks old, which is relatively early compared with the 15–22 weeks of gradual transition when piglets are reared under semi-natural conditions [1]. At weaning the piglets must adapt to a new environment with new littermates and are exposed to a sudden withdrawal of sow milk and introduced to a less digestible vegetable-based diet. The early post-weaning period is associated with stress, low feed intake and poor performance [2,3,4]. As a consequence, rapid changes in the structure and function of the small intestine (SI) are observed in the immediate post-weaning period. These changes include shortening of the villous and increase in crypt depth in the SI [5,6] as well as reduced activity of the brush border enzymes [7,8,9,10] resulting in a decline of the digestive and absorptive capacity of the SI [11]. Due to these changes, the immediate post-weaning period is associated with intestinal disturbances such as diarrhoea and depression of the growth performance [11]. It is common practice to offer piglets a dry creep feed while suckling to make the piglets familiar with solid feed prior to weaning and to prepare the gut for vegetable ingredients. A positive correlation has been found between the pre-weaning feed intake and the feed intake and growth in the immediate post-weaning period [12]. However, studies report that up to 70% of piglets are categorized as non-eaters as they fail to consume dry creep feed prior to weaning [13]. At weaning, these non-eater piglets will experience a large nutritional change when changing from sow milk to solid feed [14], which may result in post-weaning diarrhoea (PWD). Therefore, it is important to develop feeding strategies that stimulate the creep feed intake in suckling piglets and thus increase the number of eaters within a litter. The few studies that have investigated the effect of feeding liquid feed prior to weaning reported that offering creep feed in liquid, compared with dry form, increased the pre-weaning feed intake [15,16,17]. Based on these findings, it is reasonable to assume that more piglets will be categorized as eaters when offered a liquid diet. To date, no studies have investigated the distribution in the number of eaters and non-eaters when offered a liquid creep feed during the suckling period.

The aim of this study was to compare the feed intake and the number of eaters when piglets were offered either dry- or liquid creep feed prior to weaning, and how the dietary treatment and eating category affected the prevalence of PWD in an on-farm study. Furthermore, the objective was to examine how the activity of digestive enzymes were affected by the dietary treatment and eating behavior. The hypothesis was that litters fed the liquid diet during the suckling period would have a greater feed intake and more piglets within the litters would be categorized as eaters than litters fed the dry creep feed. Furthermore, it was hypothesized that the digestive enzyme activity and the prevalence of PWD would increase and decrease, respectively, in eaters compared to non-eaters.

## 2. Materials and Methods

### 2.1. Animals and Experimental Design

The experiment was conducted on-farm in a commercial Danish pig herd and included a total of 347 piglets from 29 litters (Duroc × (Danish Landrace × Yorkshire), DanBred, Denmark). The parity of the sows ranged from 1–7. The experiment included two batches with 15 and 14 litters, respectively. The piglets in batch 1 were born at day 0 ± 2 days, whereas the piglets in batch 2 were born at day 0 ± 3 days the following week. All liveborn piglets were individually weighed within 24h after the sow had finished farrowing and were ear tagged for identification. Due to standard procedures in the herd, all piglets were injected with 0.2 mL Vetrimoxin^®^ L.A. (Ceva Animal Health A/S, Vejle, Denmark) after birth and with 0.1 mL Zactran^®^ (MERIAL, Lyon, France) on day 4 postpartum in order to prevent respiratory diseases. Three days after birth all pigs received an intramuscular injection with 300 mg Fe (iron4u, Hobro, Denmark) and an oral dose of 0.4 mL Cevazuril^®^ (Ceva Animal Health A/S, Vejle, Denmark) to prevent diarrhoea caused by coccidiosis.

The piglets at litter equalization were allocated to sows depending on their size, as this was standard herd procedure; thus the smaller (<1 kg) piglets were allocated the same sows and the larger (>1.5 kg) piglets were allocated the same sows during cross fostering, respectively. At day 10, litters were fixed, and no piglets were moved either within or between dietary treatment groups. All litters consisted of 10–14 piglets. If a piglet had to be removed because of health or other issues, it was moved to a sow outside the trial, and the date and cause was noted.

At day 10, litters were allocated to one of the two dietary treatments, dry creep feed (DCF) or liquid creep feed (LCF) and balanced for size of the piglets (small or large) in order to ensure that smaller and larger piglets were equally distributed between the two treatments. The two dietary treatments offered had identical nutrient composition and only differed in physical form. The ingredients and nutrient composition of the meal diet can be found in Table 1.

Fourteen litters (*n* = 162) were offered a commercial dry creep feed diet containing 1% red ferric oxide (Borup Kemi, Denmark) as dye from day 10. The remaining 15 litters (*n* = 185) were offered the same creep feed containing 1% red ferric oxide, and mixed with water in a 1:1.5 feed-to-water ratio (36.1% dry matter) right before it was fed to the piglets at room temperature. The feed was provided in a plastic creep feeder (Hatting, Denmark) that was fixed to the slats in the floor next to the nipple drinker within each pen. Creep feed was offered 9 times a day in one-hour intervals from 0800 to 1600 h, thus the pigs had access to feed from 0800 to 1700 h. This time frame was decided upon due to previous observations on piglet feeding behavior [18] and in order to keep the liquid feed fresh. The creep feeders were manually cleaned every hour and new feed was provided. The feed disappearance was determined on a litter basis every hour by collecting and weighing the leftovers.

The piglets were, on average, weaned at day 28. According to herd practice, the piglets were weaned at different ages (ranging from 27 to 31), therefore, all piglets were individually weighed at day 24. The remaining piglets of the litters were weighed individually again at day 35 and 61 in the weaner unit.

### 2.2. Eaters and Non-Eaters

On days 18, 21, and 24 faecal swabs from each piglet were taken using cotton swabs that had been dipped in water just before sampling. The color of the swabs was determined visually immediately after sampling. Categorization of the creep feed intake of the individual piglet was based on whether dye was visible on the cotton swabs or not. On each sampling day, every piglet was categorized as an “eater” or a “non-eater” based on the color of the swabs. A red color indicated that the piglet had consumed creep feed. Piglets were categorized according to the method described by Bruininx et al. [19], where piglets with red dye on the cotton swabs on all three sampling days were categorized “good eaters”, and piglets with dye 1–2 times were categorized “moderate eaters”, while piglets that never showed dye on the cotton swabs were categorized “non-eaters”.

### 2.3. Housing and Management

At day 112 of gestation, sows were moved to the farrowing unit, consisting of 54 pens divided in 2 sections. All pens measured 2.60 m × 1.60 m. The floor consisted of 1/3 slatted floor and 2/3 solid floor. In the corner of each pen was a covered creep area with a heating lamp (175 W). All piglets had ad libitum access to water via a nipple drinker within each pen. The temperature in the farrowing unit was maintained at 24 °C during the entire lactation period.

At weaning the pigs were mixed between treatments and allocated into pens within the weaner unit. The weaner unit consisted of 3 sections with 8 pens in each section. The pigs were housed in pens with 30–38 pigs per pen. Every pen measured 5.0 m × 2.7 m. One quarter of the floor was slatted while 3/4 was solid. At one side of each pen there was a covered area measuring 1.20 m × 2.70 m. Each pen had a feeding and drinking trough measuring 1.40 m in total. All pigs were fed a dry diet ad libitum. The post-weaning feed was similar in composition to the pre-weaning feed, with the exception that for the first 10 days post-weaning, zinc was added to the diet (2500 ppm). The temperature in the weaner unit was 24 °C at trial initiation and was gradually lowered to reach 18 °C at the end of the trial. When the pigs reached approximately 30 kg, they were removed from the weaner unit. If the farm staff treated a pig for diarrhoea (Linco-Spectin, Zoetis, Parsippany, New Jersey) this was recorded as a diarrhoea treatment.

### 2.4. Tissue Sampling

Before weaning, a total of 40 piglets (20 from each batch) were euthanized on day 25 or 26 based on if they were eaters or non-eaters. Ten pigs categorized as non-eaters from the DCF groups (no dye on cotton swabs) and 10 non-eaters from the LCF group were euthanized. In batch 1, no good eaters (dye on cotton swabs 3 times) were identified in the LCF groups and only 4 were identified in the DCF groups, while 5 moderate eaters (dye on cotton swabs 2 times) from the LCF groups were euthanized, as was one pig from the DCF groups that had been categorized as an eater twice, and the remaining were good eaters (dye on cotton swabs 3 times).

The pigs were euthanized with a captive bolt pistol and exsanguinated for immediate collection of blood for plasma and serum. The EDTA tubes (BD-Plymouth, Plymouth, UK) were kept on ice until centrifuged. The serum tubes were kept at RT and centrifuged within four hours after sampling (CM-6MT; Elmi, Riga, Latvia). The EDTA tubes were centrifuged at 2000× *g* for 15 min and the serum tubes were centrifuged at 1700× *g* for 12 min, before the plasma and serum were collected. Subsequently the samples were frozen at −20 °C until further analysis. The entire gastrointestinal tract was extracted, and weight of the stomach, small intestine and colon was recorded both with and without contents. The small intestine was placed on a dissection table and length was recorded in a relaxed state. Tissue samples were collected from the proximal, medial and distal part of the small intestine and were immediately frozen on dry ice and subsequently transferred to −80 °C until later analysis of enzyme activity.

### 2.5. Analysis of Enzyme Activity

The mucosal activity of the disaccharidases; lactase, maltase, and sucrase in the proximal part of the small intestine from the 40 euthanized pigs was analyzed. First, the samples were sliced into small pieces and homogenized in 1.0% Triton-X 100 solution to break down the cell membranes and free the enzymes. The solutions were homogenized 1 min on GentleMACS, and were thereafter centrifuged at 5000× *g* for 10 min at 4 °C. The samples were frozen (−18 °C) until further analysis. At the day of analysis, the homogenates were thawed, vortexed and centrifuged at 5000× *g* for 2 min at 4 °C. The homogenates were diluted in Millipore water and transferred to 96-well microtiter plate and mixed with their specific substrates (lactose, maltose, sucrose). The microtiter plate was incubated for 30 min in the ELISA reader (SpectraMax iD3, VWR International, Radnor, PA, USA). After incubating, a 250-μL PGO color solution was added, and the plate was run for 30 min at 37 °C at 450 nm [20]. The enzyme activity was expressed as units per gram of tissue.

### 2.6. Calculations and Statistical Analysis

To compare the feed disappearance between the two dietary treatments, the feed intake was calculated on a dry matter (DM) basis. From the chemical analysis it was found that the dry diet contained 90.3% DM. The DM in the liquid diet, when mixed with water in a 1:1.5 ratio, was calculated to 36.1% DM. As the number of piglets in the litters differed, the DM disappearance was calculated as DM disappearance per piglet per litter, assuming that all pigs within the litter consumed the same amount of creep feed.

Data were analyzed using R studio version 1.1.383. The test parameters were eating category, BW, average daily gain (ADG), organ weight, DM disappearance, number of diarrhoea treatments, enzyme activity and blood metabolites. In the models for BW, eating category and DM disappearance, data were analyzed using linear mixed effects models and repeated measures were used, with day as the repeated subject. For ADG, enzyme activity, blood metabolites, organ weight and diarrhoea treatments, data were analyzed using linear models. Model reduction was performed using ANOVA, where systematic effects with a significance level of *p* > 0.1 were excluded from the model. Least square means from the models were extracted with the function Emmeans and are presented with the standard error of the mean (±SEM).

Data were analyzed according to the following model:

Yijkl = μ + αi + βj + (αβ)ij + θk + δl + εijkl

where Yijkl is the response variable and μ is the overall mean, αi denotes the systematic effect of eating category (I = good, moderate, non-eater) and βj is the systematic effect of dietary treatment (j = dry, liquid), (αβ)ij is the interaction between eating category and dietary treatment and were included when significant, θk is the random effect of sow and δl is the covariate for birth weight for the individual piglet (*n* = 1,…, 347) while εijkl ~ N(0,σ2) denotes the random error component.

## 3. Results

From day 10, the experiment included 347 piglets born from 29 sows from two batches. Batch 1 contained 179 piglets while batch 2 contained 168 piglets. Two nurse sows were used for LCF litters and one nurse sow was used for one DCF litter. At day 10 the DCF litters contained on average 11.6 piglets (ranging from 9–14), whereas the LCF litters contained 12.3 piglets on average (ranging from 10–14). Before weaning, three piglets from the DCF group died while 9 piglets from the LCF group died, mainly due to crushing by the sow. Three piglets from each group were moved to a sow not included in the trial, due to health issues.

### 3.1. Dry Matter Disappearance Pre-Weaning

The DM disappearance of each litter was measured daily from days 10–24 postpartum. For the piglets fed the DCF diet, the DM disappearance in the entire creep feeding period from day 10 to day 24 ranged from 6 g to 95 g with an average of 25 g DM per piglet in total. In comparison, the piglets fed the LCF diet had a DM disappearance ranging from 28 g to 177 g per piglet with an average DM disappearance per piglet of 45 g. The average DM disappearance per pig is listed in Table 2.

**Figure 1:**
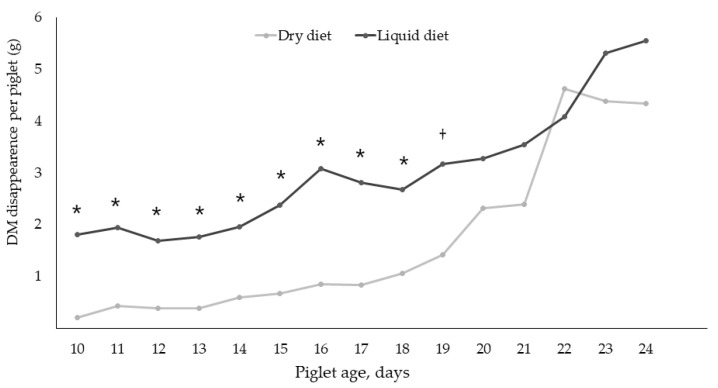
The average daily dry matter (DM) disappearance per piglet in litters fed either dry (DCF) or liquid creep feed (LCF) from day 10–24 postpartum. * Statistical significance between dietary treatments (*p* < 0.05) and † statistical tendency between dietary treatments (*p* < 0.1).

The differences in DM disappearance were highly significant (*p* < 0.001) between dietary treatments from day 10–18 where it was higher in the LCF litters. In general, the disappearance of DM increased over time (Figure 1). As seen in the figure, the DM disappearance differed between dietary treatments from day 10–19.

### 3.2. Eating Categories

A total of 330 pigs were examined all three days: 18, 21 and 24 after farrowing. Of the DCF pigs examined, 68% were categorized as eaters at least once, while 53% of the LCF pigs were categorized as eaters at least once (*p* = 0.181). Pigs were divided into eating categories, and the percentage of good eaters, moderate eaters and non-eaters between treatments did not differ (Table 3).

**Figure 2:**
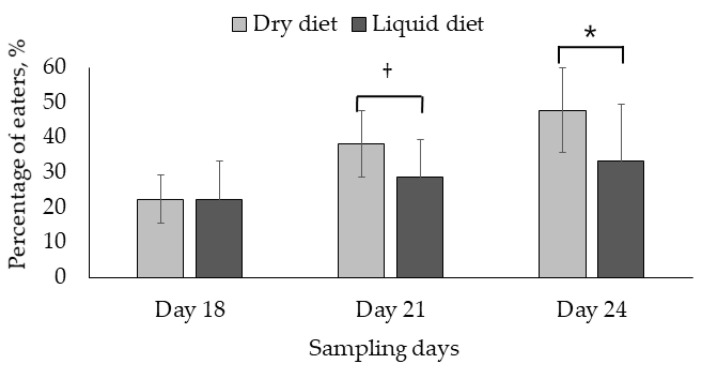
The percentage of piglets fed a dry- or liquid creep diet, categorized as eaters during the three different sampling days. * Statistical significance between dietary treatments (*p* < 0.05) and † statistical tendency between dietary treatments (*p* < 0.1).

The number of pigs categorized as eaters increased during the three sampling days (Figure 2). The increase in the number of eaters differed from day 18–21 and day 18–24 for DCF pigs (*p* < 0.001), respectively, and for LCF pigs (*p* < 0.05) day 18–24. The percentage of eaters between the two dietary treatments tended to differ at day 21 and differed at day 24 (*p* = 0.083 and *p* < 0.05, respectively).

### 3.3. Growth Performance

All pigs included in the experiment were weighed individually at birth and on day 10, 24, 35 and 61 after farrowing. The growth performance and ADG of the pigs in both treatments are listed in Table 4. The BW in the pre-weaning period did not differ between the two dietary treatments (*p* > 0.05). However, the DCF piglets had a greater ADG in the entire pre-weaning period day 0–24 than the pigs in the LCF litters (200 g/day vs. 176 g/day, *p* < 0.01). Even though the DCF pigs had a greater ADG, it was first at day 61 postpartum that the DCF pigs had a significantly greater BW than the LCF pigs (21.6 kg vs. 19.7 kg, *p* < 0.01). The DCF pigs categorized as good eaters had a greater BW at day 61 postpartum than the DCF pigs categorized as non-eaters (23.1 kg vs. 20.2 kg, *p* < 0.05).

### 3.4. Organ Weights

Ten piglets categorized as eaters and ten piglets categorized as non-eaters from each dietary treatment were euthanized before weaning. Of the 40 piglets sampled, the content of the stomach of one of the non-eaters was clearly red from the dye, which is why this piglet was categorized as an eater instead of non-eater, and another non-eater was selected. The BW of the DCF pigs at the day of euthanization were greater (6.69 kg vs. 5.84 kg, *p* < 0.05) than the LCF pigs, and as the BW differed between dietary treatments, relative weights are also presented (Supplementary Table S1). The relative weight of the full SI and full colon were greater (*p* < 0.05) for the LCF pigs than the DCF pigs. The relative weight of the empty SI and empty colon of the LCF pigs tended to be heavier as well (*p* = 0.056 and *p* = 0.060, respectively). While the relative weight of the empty stomach was similar between treatments.

### 3.5. Enzyme Activity

The activity of lactase, maltase and sucrase were measured in the proximal part of the small intestine of the 40 pigs euthanized. The enzyme activity was different between dietary treatments (*p* < 0.01) with the DCF pigs displaying the greatest activity of all 3 disaccharidases (Table 5). No effect of the eating category was found with respect to the activity of any of the disaccharidases (*p* > 0.05).

### 3.6. Blood Serum Metabolites

The metabolites analyzed from the blood samples are listed in Supplementary Table S2. Effects of the dietary treatment were found for the levels of alanine aminotransferase where the levels were highest for the LCF pigs (*p* < 0.05). Tendencies towards effects of dietary treatment were found for the levels of cholesterol, creatinine, and inorganic phosphate where the DCF had higher levels of all three (*p* = 0.1). The pigs categorized as eaters had a greater amount of creatine kinase (*p* = 0.023) and tended to have a greater amount of aspartate aminotransferase in the blood (*p* = 0.054), whereas the pigs categorized as non-eaters tended to have a higher amount of blood urea nitrogen (*p* = 0.079).

### 3.7. Post-Weaning Diarrhoea Treatments

A total of 290 pigs were weaned, and out of these 11 pigs were treated for PWD. Dietary treatment during the pre-weaning period tended to affect the occurrence of PWD with 3 pigs fed the LCF pre-weaning, and 8 pigs fed the DCF pre-weaning were treated for diarrhoea (*p* = 0.076). Of the pigs treated for diarrhoea, 7 pigs were categorized as non-eaters and 4 pigs were categorized as moderate eaters. No pigs categorized as good eaters before weaning were treated for PWD. A tendency for the eating category (good, moderate, non-eater), was found on pigs treated for PWD as more non-eaters were treated than the other eating categories (*p* = 0.073). The percentage of non-eater pigs treated for PWD was different between dietary treatments, with more pigs fed the dry diet treated (*p* = 0.021).

## 4. Discussion

Feed disappearance between litters can vary greatly, as reported in earlier studies (e.g., [13,19]). In the present study, the feed disappearance of the pigs offered the dry diet ranged from 7 g to 125 g per piglet for the entire creep feed period with an average of 33 g in total. A similar level of feed intake was observed by Collins et al. [21] who reported an average feed consumption per pig of approximately 34 g in total, when fed a complex dry creep diet from day 9–22. Another group of pigs in the same study were offered a simple dry creep diet, but in this case, they had a greater feed consumption with an average of 48 g per pig in total [21]. Bruininx et al. [19] reported an average feed intake of a dry pelleted diet to be 301 g per piglet in total, and Bruininx et al. [22] reported the average feed intake in the creep feeding period from day 11–28 to be a total of 377 g per pig. These feed intakes are much greater than what was found in the present study, which might be explained by the fact that in Bruininx et al. [19,22] the pigs had four days more to consume the feed due to longer pre-weaning period, whereas 60–65% of the feed was consumed from day 22–28 in these studies. Furthermore, in the previous studies the pigs had access to feed 24 h a day, whereas in the present study pigs had access to creep feed from 0800 to 1700 h. As it was not possible to provide fresh liquid creep feed for 24 h due to on-farm limitations, the daily period of feed provision was based on the study by Jakobsen et al. [18] reporting that pigs visit the feeder more frequently during this interval, and thus increasing the chance of greater feed consumption. Toplis et al. [17] reported a DM intake in the pre-weaning period from day 14–24 to be a total of 91 g DM per pig when fed a dry pelleted diet, and 374 g DM per pig when fed a liquid diet (1:2 feed-to-water ratio) where the feed was available 24 h a day. In the present study, the total DM disappearance of the piglets fed the liquid diet was on average 45 g per pig in the entire creep feeding period and 25 g per pig for the dry diet. When taking into account the hours the pigs did not have access to feed in the present study, the DM disappearance is still relatively small compared to the previous studies.

Numerically, more pigs fed the dry diet were moderate- and good eaters and fewer were non-eaters than the pigs fed the liquid diet, and our hypothesis is therefore rejected. Pigs offered the liquid diet did show a greater DM disappearance up until day 18 than the pigs fed the dry diet, and it was therefore expected that the liquid diet increased the number of eaters. However, either the pigs that were eaters have consumed more of the liquid- than the dry diet, or more pigs than could be categorized as eaters have consumed feed, but not enough to dye the faeces so that it could be recognized on the swabs. The 15h without access to creep feed may have masked the dye in the faeces, making it hard to recognize the eaters. However, part of the greater DM disappearance in LCF litters may also be due to wastage from the troughs as the pigs walked a lot in the creep feeders in the beginning of the creep feeding period, contributing to feed wastage and thereby feed disappearance.

As the DM disappearance increased from the first day of faecal swabs (day 18) to the second and third days of swabs (day 21 and 24), it was expected that the number of eaters would increase as well. This was also the case, but the number of eaters in the DCF litters increased more than the number of eaters in the LCF litters, even though the LCF litters had a greater DM disappearance. From day 20, the DM disappearances were similar between dietary treatments, which may be why no more pigs from the LCF litters were categorized as eaters. Thus, it would be expected that the number of eaters would be similar between dietary treatments the last two sampling days, which was not the case.

The number of non-eaters in the present study is high compared to other studies. Bruininx et al. [19,22] reported 19% and 10% non-eaters on a pelleted dry diet, respectively. However, the faeces of about 1/3 of the pig were impossible to judge and data from these pigs were not included in the analysis [19,22]. In the present study, all pigs were categorized into an eating category. If the color was doubtful, the pig was considered a non-eater at sampling, which may partly explain the major difference in the number of non-eaters between this and the studies of Bruininx et al. [19,22]. Furthermore, the pigs in Bruininx et al. [19,22] were tested at day 18, 22 and 27, which is later than in the present study, which also may contribute to more eaters, as the feed intake increases with age and the greater feed intake makes identification by the dye on the swabs easier. In the study by Collins et al. [21] the feed intake of the complex diet was similar to the feed disappearance of the dry diet in the present study. However, even though the days of sampling were earlier, more good eaters and less non-eaters were categorized in that study compared to the present study. Ferric oxide was chosen as the coloring dye in the current study due to the study being on-farm, however, this dye has not been used to determine eaters and non-eaters before, and therefore the inclusion rate of the dye in the feed was possibly insufficient. One percentage of ferric oxide was added to the feed before water was added, which was based on several studies using one percent chrome (III) oxide [19,22,23,24]. As early as 1989, Barnett et al. [25] questioned the method of faecal swabs, suggesting that a large milk intake may mask the dye in the faeces. This may also be the case for the water added to the diet, and it may be that more dye should have been added to the liquid diet, due to dilution by the water. In one of the LCF litters, all pigs were categorized as non-eaters. However, this litter had a feed disappearance of 1113 g (392 g DM) during the creep feeding period. This suggests that either the pigs have not consumed the feed but had a great feed wastage, or that the method of faecal swabs were insufficient to find the eaters.

In most of the eaters euthanized, the contents of colon and the SI only showed weak signs of dye. The retention time of the entire gastrointestinal tract for 14-day old piglets is 41 h for liquid milk diets and 25h for pelleted diets [26], while the retention time in 35 days old piglets is slightly longer and ranges from 34 to 44 h [26]. The pigs in the present study were 25 and 26 ± 3 days old, which is why the retention time would be expected to be in between the ranges mentioned above. The last sample of swabs were taken at day 24, and the pigs were euthanized on days 25 and 26. Some of the non-eaters may have started the consumption of creep feed within this time frame. This was the case for at least one of the pigs. The content of the stomach was clearly red, which is why this pig was categorized as an eater instead of non-eater.

### 4.1. Maturation of the Gastro-Intestinal Tract

Brush border digestive enzymes such as maltase, lactase and sucrose all increase throughout the suckling period, preparing and maturing the gut towards being capable of hydrolyzing more complex carbohydrates than lactose and are important in order to prepare the gut towards more adult-type plant-based diets. Typically, dry creep feed is offered to piglets before weaning to prepare the SI for an increasing percentage of vegetable ingredients as well as familiarizing the piglets with solid feed. This may partly explain why it can be beneficial, in regards to the subsequent weaning process, to offer solid food (creep feed) to suckling pigs as it may accelerate gut maturation by increasing first the production of salivary amylase, to the downstream production of disaccharides. Consistent with this, a positive correlation has been found between pre-weaning feed intake and subsequent feed intake and growth in the immediate post-weaning period [12]. The enzyme activity was greater in DCF pigs than in LCF pigs, suggesting a higher digestive capacity of the proximal part of the small intestine in these pigs. The lower enzyme activity in the LCF pigs, even with the higher DM disappearance, indicates that the substrate itself also influences enzyme activity. No difference in the enzyme activity was found between eating categories, which may be due to the very low feed intake of the litters, and the uncertainty of the method of faecal swabs. It is not clear that pigs categorized as non-eaters did not consume creep feed at all, and it is unknown how much the pigs categorized as eaters consumed. As the enzyme activity of the non-eaters differed between dietary treatments, it indicates that at least some of them consumed creep feed. We had expected the non-eaters to have the same levels, but in hindsight a third group of piglets with no access to creep feed should have been included as a negative control group.

The higher enzyme activity of lactase in the DCF pigs may reflect that these pigs have consumed more sow milk than the LCF pigs. In contrast, Hampson and Kidder [27], found a significant decline in the lactase activity in unweaned pigs with access to creep feed compared to pigs with no access to creep feed. The mechanisms behind the greater enzyme activity in the DCF pigs are not clear, but as the activity of sucrase and maltase increases from birth and throughout the suckling period [28], it might indicate gut maturation. However, it may also be because of a greater creep feed intake, as Kelly et al. [29] reported numerically higher activities of sucrase and maltase in piglets fed a high amount of creep feed compared to piglets fed a lower amount or no creep feed. To the best of the authors knowledge, no previous studies comparing the effects of liquid- and dry- feeding during suckling on the activity of the digestive enzymes have been published.

### 4.2. Post-Weaning Diarrhoea Treatments

A tendency of an effect of the dietary treatment on the prevalence of PWD treatments was found, but this was attributed to the non-eaters. More DCF non-eater pigs were treated for PWD than LCF non-eater pigs. The number of non-eater pigs treated were expected to be similar between dietary treatments as these pigs should only have consumed sow milk, but the results indicate that other factors affected the occurrence of PWD besides eating category and dietary treatment. A tendency was found that more non-eaters were treated for PWD than the other eating categories, which suggests that non-eaters are more susceptible to PWD than eaters. However, as only 11 pigs were treated for PWD it is not possible to draw any conclusions. Previous studies comparing the prevalence of PWD in eaters and non-eaters are very limited, and do not report any difference between the eating categories and the prevalence of PWD [30].

## 5. Conclusions

In conclusion, the experimental design had some limitations due to it being an on-farm study. We did not successfully identify eaters and non-eaters and can therefore not conclude on gut maturation between eaters and non-eaters. However, we did find an increase in the activity of lactase, sucrase and maltase in the DCF group suggesting a more mature gut in these pigs compared with the LCF group. In line with this observation, the DCF pigs had a greater BW at day 61 postpartum, indicating that the DCF pigs adapted more easily to the weaning phase. However, both observations were in contrast to what was expected, as previous studies have indicated that liquid compared with dry creep feed elicits greater feed intake and growth. For future studies, individual housing with the possibility of exact assessment of feed intake is recommended.

## Figures and Tables

**Table 1:** Ingredients and composition of the dry meal creep feed offered to the piglets from day 10–24 postpartum.

Ingredients, %	Batch 1	Batch 2
Wheat	47.0	
Barley	16.0	
Soy protein concentrate	13.0	
Milk powder	8.0	
Fish meal	4.0	
Heated wheat	4.0	
Vitamin/mineral premix ^1^	2.7	
Potato protein concentrate	2.5	
Vegetable oil	2.0	
Calcium formate	0.6	
Nutri Novo ^2^	0.2	
Chemical analysis		
Energy, MJ/kg	16.4	16.2
Dry matter, %	90.4	90.1
Crude protein, %	18.8	19.1
Crude fat, %	4.2	4.2
Ash, %	6.7	6.8
Calcium, %	0.807	0.878
Phosphorus, %	0.644	0.665
Lysine, %	1.41	1.31
Threonine, %	0.748	0.780
Methionine, %	0.400	0.417
Cysteine + Cystine, %	0.300	0.288

^1^ Nutrients added per kilogram of feed: A vitamin; 12,000 IU, D3- vitamin; 1200 IU, E-vitamin dl-alpha-tocopherol; 180 mg, Ferric(II)-sulphate monohydrate; 90 mg, Ferric (II)—fumarate 90 mg, Copper(II)-sulphate pentahydrate; 140 mg, Manganese(II)-oxide; 53 mg, Zinc oxide; 132 mg, Calcium iodate 0.3 mg, Natrium selenite; 0.35 mg. ^2^ Mono-, di- and triglycerides.

**Table 2:** Average dry matter (DM) disappearance per pig for litters fed a dry (DCF) or liquid creep feed (LCF) day 10–24 postpartum.

	Treatment			
DM Disappearance, g	DCF	LCF	SEM	*p*-Value
Day 10–18	5.43	20.07	2.17	<0.001
Day 18–21	7.19	12.66	2.90	0.193
Day 21–24	15.73	18.48	5.66	0.734
Day 10–24	24.89	44.98	8.65	0.108

**Table 3:** Percentage of good eaters, moderate eaters and non-eaters in piglets receiving either dry (DCF) or liquid creep feed (LCF). Categorization was based on faecal swabs day 18, 21 and 24 postpartum with good and moderate eaters categorized as eaters on 3 or 1–2 samplings days, respectively, where non-eaters were the pigs never categorized as eaters on either of the sampling days.

	Treatment			
	DCF	LCF	SEM	*p*-Value
*n*	156	174		
Good eaters	10% (15)	9% (15)	2.6 (0.30)	0.973
Moderate eaters	58% (91)	44% (78)	5.1 (0.56)	0.131
Non-eaters	32% (50)	47% (81)	5.8 (0.67)	0.182

**Table 4:** Average BW and ADG of pigs fed a dry (DCF) or liquid (LCF) creep feed from day 10–24. After weaning all pigs were fed a dry diet.

	Treatment			
BW, kg	DCF	LCF	SEM	*p*-Value
Day 0	1.21	1.57	0.454	0.364
Day 10	2.43	2.63	0.454	0.688
Day 24	6.01	5.70	0.454	0.538
Day 35	9.15	8.41	0.466	0.162
Day 61	21.6	19.7	0.509	0.003
ADG, g/day				
Day 0–10	128	109	0.005	0.099
Day 10–24	251	224	0.007	0.031
Day 0–24	200	176	0.006	0.003
Day 24–35	272	249	0.014	0.208
Day 35–61	468	440	0.016	0.304
Day 24–61	411	378	0.013	0.087

**Table 5:** The enzyme activity in the proximal part of the small intestine in pigs fed a dry (DCF) or liquid (LCF) diet and categorized into eating categories based on 3 fecal swabs. Eaters are defined by the presence of feed dye on 2–3 swabs whereas non-eaters never showed dye on the swabs.

	Treatment						
	DCF		LCF		SEM	*p*-Value ^1^	
	Eater	Non-Eater	Eater	Non-Eater		Treatment	Eating Category
*n*	10	10	10	10			
Maltase, U/g	16.9 ^a^	16.9 ^a^	11.0 ^b^	10.9 ^b^	1.54	0.004	0.994
Sucrase, U/g	7.39 ^a^	7.13 ^a^	3.51 ^b^	3.25 ^b^	0.64	<0.001	0.712
Lactase ^2^, U/g	24.9 ^a^	25.8 ^a^	13.6 ^b^	14.5 ^b^	2.27	0.001	0.722

^a,b^ Means in the same row with different superscripts differ significantly (*p* < 0.05). ^1^ No interactions between treatment and eating category were observed. ^2^ The activity of lactase in two non-eater DCF pigs and one LCF eater pig could not be measured, and the lactase activity is therefore based on data from 37 pigs.

## Data Availability

Data is available on request.

## Supplementary Materials

The following are available online at https://www.mdpi.com/article/10.3390/ani11113144/s1, Supplementary Table S1: The average BW, weight of the stomach, small intestine (SI) and colon both full and empty of 40 pigs euthanized at day 25 or 26. The pigs were fed either a dry- or liquid creep diet from day 10 and categorized as eaters or non-eaters based on the color of faecal swabs. The weight of SI, stomach and colon are both measured as absolute and relative weight (% of BW). Supplementary Table S2: Blood parameters from 40 piglets fed a dry (DCF) or liquid creep feed (LCF) during suckling and categorized as eaters or non-eaters based on faecal swabs.
