# Supplementary material for: The Effect of Feeding Liquid or Dry Creep Feed on Growth Performance, Feed Disappearance, Enzyme Activity and Number of Eaters in Suckling Piglets
Source: Animals (Basel). 2021 Nov 4;11(11):3144. doi: 10.3390/ani11113144 (PMC8614247; doi:10.3390/ani11113144)
Supplement: Supplementary file 1 [file animals-11-03144-s001.zip › animals-1386418-supplementary.pdf]

animals

MDPI

Article

The Effect of Feeding Liquid or Dry Creep Feed on Growth Performance, Feed Disappearance, Enzyme Activity and Number of Eaters in Suckling Piglets

Nanna Byrgesen [1], Johannes G. Madsen [1], Christina Larsen [1], Niels J. Kjeldsen [2], Malene S. Cilieborg [1] and Charlotte Amdi [1,*]

[1] Department of Veterinary and Animal Sciences, Faculty of Health and Medical Sciences, University of Copenhagen, Grønnegårdsvej 2, 1870 Frederiksberg, Denmark; nanna.byrgesen@gmail.com (N.B.); johannes.g.madsen@sund.ku.dk (J.G.M.); christinalarsen@sund.ku.dk (C.L.); macilie@sund.ku.dk (M.S.C.); ca@sund.ku.dk (C.A.)
[2] SEGES Danish Pig Research Centre, Axeltorv 3, 1609 Copenhagen, Denmark; njk@seges.dk (N.J.K.)
* Correspondence: ca@sund.ku.dk

Supplementary Table S1: The average BW, weight of the stomach, small intestine (SI) and colon both full and empty of 40 pigs euthanized at day 25 or 26. The pigs were fed either a dry- or liquid creep diet from day 10 and categorized as eaters or non-eaters based on the colour of faecal swabs. The weight of SI, stomach and colon are both measured as absolute and relative weight (% of BW).

	Treatment				SEM	*P*-value	
	DCF		LCF				
	Eaters	Non-eaters	Eaters	Non-eaters		Treatment	Eating category
No. of pigs	10	10	10	10			
BW, kg	6.69[a]	6.70[a]	5.84[b]	5.85[b]	0.330	**0.046**	0.984
Stomach full, g	119	113	139	133	14.2	0.247	0.709
Stomach full, %	1.87[x]	1.74[x]	2.41[y]	2.28[y]	0.229	**0.066**	0.608
Stomach empty, g[1]	46.7[ax]	40.2[x]	34.5[by]	39.6[y]	3.21	**0.079**	0.382
Stomach empty, %	0.66	0.65	0.65	0.64	0.041	0.849	0.890
SI full, g	285	268	270	335	2.41	0.333	0.323
SI full, %	4.02[a]	4.40[a]	5.02[b]	5.40[b]	0.264	**0.005**	0.195
SI empty, g	244	229	211	254	1.63	0.797	0.379
SI empty, %	3.46[x]	3.71[x]	3.90[y]	4.14[y]	0.177	**0.056**	0.215
SI length, m	8.53	8.77	8.53	8.77	0.219	0.996	0.329

Colon full, g	121	118	124	121	7.90	0.738	0.735
Colon full, %	1.84[a]	1.80[a]	2.15[b]	2.10[b]	0.111	**0.034**	0.737
Colon empty, g	66.4	67.8	66.0	67.3	3.14	0.919	0.746
Colon empty, %	1.02[x]	1.03[x]	1.16[y]	1.17[y]	0.058	**0.060**	0.854

[a,b] Means in the same row with different superscripts differ significantly (*P*<0.05)

[x,y]Means in the same row with different superscripts tended to differ (*P*<0.1)

[1]Interaction was found between eating category and dietary treatment (*P*=0.012)

Supplementary Table S2: Blood parameters from 40 piglets fed a dry (DCF) or liquid creep feed (LCF) during suckling and categorized as eaters or non-eaters based on faecal swabs.

	Treatment				SEM	*P*-value	
	DCF		LCF				
	Eaters	Non-eaters	Eaters	Non-eaters		Dietary treatment	Eating category
n	10	10	10	10			
Albumin, g/L	34.3	31.8	31.3	33.2	1.40	0.556	0.810
Total protein, g/L	47.7	48.4	47.0	47.9	1.31	0.602	0.696
Alanine aminotransferase, U/L	41.6[a]	36.3[a]	52.0[b]	46.7[b]	3.16	**0.012**	0.134
Basic phosphatase, U/L	877	762	984	869	94.9	0.335	0.301
Total bilirubin, umol/L	5.0	5.0	6.1	6.1	0.76	0.289	0.977
Creatin Kinase, U/L	8234[a]	5708[b]	6999[a]	4473[b]	934	0.305	**0.023**
Creatinine, umol/L	88.7[x]	89.2[x]	79.5[y]	80.2[y]	4.18	**0.067**	0.902
Cholesterol, mmol/L	4.4	4.0	4.0	3.5	0.25	0.182	0.112
Inorganic Phosphate, mmol/L	3.2[x]	3.1[x]	3.0[y]	2.9[y]	0.09	**0.090**	0.525
Iron, umol/L	28.3	26.6	25.1	23.4	2.44	0.291	0.536
Aspartate aminotransferase, U/L	206[x]	161[y]	213[x]	167[y]	20	0.773	**0.054**
Gamma-glutamyl transferase, U/L	13.2	12.9	16.7	16.4	3.00	0.362	0.916
Blood urea nitrogen, mmol/L	2.7[x]	3.5[y]	3.4[x]	4.2[y]	0.41	0.177	**0.079**
Calcium, mmol/L	3.2	3.3	3.2	3.2	0.18	0.845	0.796
Magnesium, mmol/L	0.98	1.00	0.95	0.97	0.060	0.664	0.814
Sodium, mmol/L	144	140	137	133	6.01	0.297	0.556

Potassium, mmol/L	7.1	6.7	6.9	6.6	0.23	0.651	0.182
Glucose, mmol/L	7.3	7.3	7.0	7.1	0.26	0.370	0.832
Triglycerides, mmol/L	1.6	1.6	1.6	1.6	0.15	0.965	0.827

[a,b] Means in the same row with different superscripts differ significantly ($P<0.05$)

[x,y] Means in the same row with different superscripts shows a statistical tendency ($P<0.1$)
